# Cardiac Transcriptome Analysis Reveals Nr4a1 Mediated Glucose Metabolism Dysregulation in Response to High-Fat Diet

**DOI:** 10.3390/genes11070720

**Published:** 2020-06-29

**Authors:** Lihui Men, Wenting Hui, Xin Guan, Tongtong Song, Xuan Wang, Siwei Zhang, Xia Chen

**Affiliations:** Department of Pharmacology, College of Basic Medical Sciences, Jilin University, Changchun 130021, China; menlh@jlu.edu.cn (L.M.); huiwt18@mails.jlu.edu.cn (W.H.); xguan_jlu@163.com (X.G.); TongtongSong2018@163.com (T.S.); wangxuan4018@163.com (X.W.); zhangsiwei2366@163.com (S.Z.)

**Keywords:** cardiac transcriptome analysis, high-fat diet, Nr4a1, glucose metabolism

## Abstract

Obesity is associated with an increased risk of developing cardiovascular disease (CVD), with limited alterations in cardiac genomic characteristics known. Cardiac transcriptome analysis was conducted to profile gene signatures in high-fat diet (HFD)-induced obese mice. A total of 184 differentially expressed genes (DEGs) were identified between groups. Based on the gene ontology (GO) term enrichment and Kyoto Encyclopedia of Genes and Genomes (KEGG) pathway enrichment analysis of DEGs, the critical role of closely interlocked glucose metabolism was determined in HFD-induced cardiac remodeling DEGs, including *Nr4a1*, *Fgf21*, *Slc2a3*, *Pck1*, *Gck*, *Hmgcs2*, and *Bpgm*. Subsequently, the expression levels of these DEGs were evaluated in both the myocardium and palmitic acid (PA)-stimulated *H9c2* cardiomyocytes using qPCR. *Nr4a1* was highlighted according to its overexpression resulting from the HFD. Additionally, inhibition of *Nr4a1* by siRNA reversed the PA-induced altered expression of glucose metabolism-related DEGs and hexokinase 2 (HK2), the rate-limiting enzyme in glycolysis, thus indicating that *Nr4a1* could modulate glucose metabolism homeostasis by regulating the expression of key enzymes in glycolysis, which may subsequently influence cardiac function in obesity. Overall, we provide a comprehensive understanding of the myocardium transcript molecular framework influenced by HFD and propose *Nr4a1* as a key glucose metabolism target in obesity-induced CVD.

## 1. Introduction

Given the typical modern-day high-calorie diet and sedentary lifestyle, obesity has become a worldwide epidemic [1,2]. Clinical and epidemiological evidence has shown that obesity is the predominant risk factor for cardiovascular disease (CVD), particularly coronary heart disease, arrhythmias, hypertension, heart failure, and sudden cardiac death [3]. Obesity is a contributor to increased morbidity and mortality, primarily from CVD. Furthermore, obesity contributes to the development of heart failure by 33% in a BMI-dependent increment [4]. Excess body weight and accumulated abdominal fat result in cardiac hemodynamic alterations. Consequently, such alterations lead to an adverse cardiac structure and ventricular function, especially an abnormal left ventricular geometry and diastolic capacity [3]. Obesity-mediated hemodynamics in conjunction with metabolic alterations and neurohormonal abnormalities are the primary contributors to accelerated cardiac remodeling outcomes [5]. In particular, growing evidence suggests that metabolic alterations occurring in obesity are causative in cardiac remodeling [6,7].

To support the high energetic requirement during cardiac muscle contractions, the cardiac metabolic network utilizes multiple substrates for ATP production, including glucose, fatty acids, and amino acids [8,9]. Obesity causes cardiac metabolism changes resulting in the inhibition of the metabolic network responsible for glucose utilization and oxidation, making ATP production and utilization less efficient [10]. Impairment in coordinated signaling pathways has been observed in essential cardiac metabolic genes via allosterism and post-translational protein modifications. Additionally, the cardiac metabolism network is generally modulated by metabolic regulators, particularly transcription factors [11,12]. Although previous studies revealed that a high-fat diet (HFD) induced significant alterations in the metabolic profile that are closely related to cardiac remodeling [13,14], detailed information on the influence on cardiac metabolism network alterations and biochemical adaptations occurring in HFD remain unclear. Systems biology approaches have been applied to elucidate the mechanisms of CVD, providing a comprehensive perspective on biological changes [15,16]. Transcriptome analysis could provide global information on gene expression profiling in HFD-induced cardiac remodeling, and the mechanism by which HFD influences the cardiac glucose metabolism network, which will serve to develop the current understanding of potential regulatory targets in obesity-induced CVD. Based on the bioinformatic analysis, the core genes in metabolic enzymes, translational factors, and metabolic intermediates could be identified.

Orphan nuclear receptor (*Nr4a1*), also known as Nur77, NGFI-B, or TR3, is a transcription factor of the Nr4a subfamily involved in a wide array of important biological processes, including stress response, metabolism, and cell apoptosis [17,18]. Nr4a1 has emerged as a therapeutic strategy for suppressing cardiac fibrosis and heart failure via transcriptional regulation of cardiomyocyte apoptosis-related genes [19]. Another study showed that translocation of Nr4a1 from the nucleus to the mitochondria in neonatal rat cardiomyocytes induced by oxidative stress could also contribute to cardiomyocyte apoptosis [20]. Recently, its central role in linking CVD to metabolic stress has been identified. *Nr4a1* serves as a glucose sensor in the adult heart and may mediate part of glucose’s effects in the stressed myocardium [21]. It negatively regulates cardiac contractility by regulating the hexosamine biosynthetic pathway and cardiac O-GlcNAcylation [22]. Additionally, *Nr4a1* regulates lipid metabolism in muscle cells by modulating the expression of lipid transporter proteins, such as CD36 [23]. However, its principal role in modulating myocardial metabolism homeostasis in response to HFD has not been fully illustrated.

In the current study, transcriptome analysis was constructed to obtain whole cardiac transcriptome profiling alterations in response to HFD-induced obesity. With the inclusion of *Nr4a1*, the primary role of several glucose-homeostasis metabolism-related genes was identified and further validated using in vivo and in vitro tests. The regulatory effect of *Nr4a1* in the metabolic adaptation of cardiac remodeling in response to HFD was investigated. Hence, our study provides a comprehensive insight into obesity-related cardiac transcription alterations that will serve to unravel the underlying mechanisms. In addition, a promising role of *Nr4a1* was illustrated as a target in the prevention of obesity-induced CVD.

## 2. Materials and Methods

### 2.1. Animals and Diets

Twenty-four 8-week-old male C57BL/6J mice were purchased from the Experimental Animal Center of Jilin University (China). All animals were housed in a barrier system under regulated temperature (21±2 °C), humidity (50±5%), and a light-dark cycle of 12 h per day. Before the initiation of the experiments, all mice were acclimatized for 7 days. Mice were randomly divided into a normal diet (ND) group and a high-fat diet (HFD) group of n = 12 mice per group. Mice in the ND group were fed standard chow (12% kcal fat, 23% kcal protein, 65% kcal carbohydrate) for eight weeks. In contrast, mice in the HFD group were fed a high-fat diet (Research Diets D12492 diet, 60% kcal fat, 20% kcal protein, 20% kcal carbohydrate) for eight weeks. Animals had free access to food and water. At the end of experimental period, biological samples were collected after mice were euthanized using carbon dioxide. All animal studies were performed according to the institutional guidelines for the care and use of laboratory animals. Protocols were approved by the Laboratory Animal Ethics & Welfare Committee at the College of Basic Medical Sciences of Jilin University (Approval Number 2018092).

### 2.2. Biochemical Analyses

Body weight was measured weekly throughout the experiment. Blood glucose was measured at the end of 8 weeks. At the end of experimental period, blood samples of the fasted mice were collected and placed on ice for 30 min. Then centrifuged at 3000 rpm for 10 min at 4 °C to get the serum samples that were kept at −80 °C for biochemical analysis. Serum triglyceride (TG) and total cholesterol (TC) contents were measured enzymatically using commercial kits (Cat#A110-1 and Cat#A111-1, Nanjing Jiancheng Bioeng. Inst.). The activities of lactate dehydrogenase (LDH, Cat#2401131) and creatine kinase (CK, Cat#22401127) were measured by commercial kits obtained from BioSino Bio-Technology & Science Inc. (Beijing, China). Three biological repeats were performed for each sample.

### 2.3. Heart Histopathology

The cardiac tissue was infiltrated into 10% formalin buffer for 24 h to dehydrate and fix the tissue. Next, the tissue was trimmed and embedded in paraffin. Sections (5 μm thick) were stained with hematoxylin and eosin (H&E) and Sirius Red staining. Circularly polarized images were obtained using Axio Imager M2 for polarized light microscopy (ZEISS, Oberkochen, Germany). H&E staining can display general morphological and structural characteristics in left ventricular myocardium. Quantitative analysis of cardiomyocyte cross-sectional area was conducted by measuring 30 cardiomyocytes from 3 views per section. In Sirius Red staining, the collagen fibers are stained with picrosirius red and viewed with polarized light, depending on the thickness of the collagen fibers. The hues range from green to yellow to orange to red. Cardiac collagen area of left ventricular myocardium was quantitated in ND and HFD groups. The mean area of collagen deposition was obtained by summation of Sirius red-positive areas on each section divided by the total numbers of sections. Image-pro Plus 6.0 software (Media Cybernetics, Silver Spring, MD, USA) was applied for quantitative analysis.

### 2.4. Total RNA Extraction and Validation

Total RNA of whole myocardial tissue was extracted using the mirVana miRNA Isolation Kit (Ambion Inc., Austin, TX, USA) following the manufacturer’s protocol, *n* = 3 mice per group. RNA integrity was evaluated using an Agilent 2100 Bioanalyzer (Agilent Technologies, Santa Clara, CA, USA). Samples with an RNA Integrity Number (RIN) ≥ 7 were subjected to subsequent analysis. The libraries were constructed using TruSeq Stranded mRNA LTSample Prep Kit (Illumina, San Diego, CA, USA) according to the manufacturer’ s instructions. These libraries were then sequenced on the Illumina sequencing platform (HiSeqTM 2500 or Illumina HiSeq X Ten), and 125 bp/150 bp paired-end reads were generated.

### 2.5. Transcriptome Data Mapping and Differential Expression Analysis

Raw data (raw reads) were processed using Trimmomatic software. The reads containing ploy-N and low-quality reads were removed to obtain clean reads. Following this, clean reads were mapped to the reference genome using HISAT2. The FPKM value of each gene was calculated using Cufflinks, and the read counts of each gene were obtained using htseq-count. Differentially expressed genes (DEGs) were identified using the DESeq (2012) R package functions to estimate size factors and the nbinom test. A *p*-value < 0.05 and fold change >2 or fold change <0.5 was set as the threshold for significantly differential expression. A hierarchical cluster analysis of DEGs was performed to explore gene expression patterns. Gene ontology (GO) term enrichment [24,25] and Kyoto Encyclopedia of Genes and Genomes (KEGG) [26] pathway enrichment analysis of DEGs were performed using R, based on the hypergeometric distribution.

### 2.6. H9c2 Culture and Treatment

The embryonic rat heart-derived cell line H9c2 was obtained from the Shanghai Institute of Biochemistry and Cell Biology (Shanghai, China). H9c2 cells were cultured in Dulbecco’s Modified Eagle’s medium (DMEM; Gibco, Eggenstein, Germany) containing 2.25  g/L glucose medium supplemented with 10% fetal bovine serum (FBS), 100  U/mL of penicillin, and 100 mg/mL of streptomycin, at 37 °C with 5% CO_2_ in humidified air. Cells between passages 4 and 8 were used for the experiments. For in vitro studies, stock solutions of 5 mM PA/10% fatty acid-free bovine serum albumin (BSA) were prepared and stored at −20 °C. Stock solutions were heated for 5 min at 65 °C and then cooled to room temperature before use. The fatty acid-BSA complex was added to the serum-containing cell culture medium to achieve a fatty acid concentration of 100 μM.

Commercial *Nr4a1* siRNA was used to inhibit NR4A1 protein expression according to the manufacturer’s instructions. Transfection of H9c2 cells with siRNAs was carried out using Lipofectamine™ 2000 (Invitrogen, Carlsbad, CA, USA), according to the manufacturer’s instructions. H9c2 cells were treated with a mixture containing DMEM, lipo2000, and *Nr4a1* siRNA for 4 h. Subsequently, the mixture was replaced with DMEM and cultured for 72 h. Specific siEGFR sequences were as follows: sense, UGGCCCAGAGUUCCCUGAAdTdT, and antisense, UUCAGGGAACUCUGGGCCAdTdT. Transfected cells were then treated with PA, as previously described.

### 2.7. Quantitative Real-Time PCR (qPCR) Validation

According to the microarray results, the glucose metabolism-related DEGs and key enzymes in glycolysis were chosen for further validation by qPCR in HFD mice and PA-induced H9c2 cardiomyocytes. Cardiac samples were collected, and total RNA was isolated using TRIzol^®^ reagent (Invitrogen, Carlsbad, CA, USA), and complementary DNA was synthesized using the *TransScript* R Frist-Strand cDNA Synthesis SuperMix (TransGen, Biotech Co., Ltd., Beijing, China) according to the manufacturer’s instructions. Primer sets for selected genes were designed by Sangon Biotech (Shanghai, China); their sequences and reaction conditions are listed in Table S1. Each sample was run in triplicate in 96-well plates using LightCycler *R* 96 and FastStart Essential DNA Green Master (Roche Diagnostics GmbH, Mannheim, Germany). Quantification cycles were calculated using the fit point method (LightCycler R 96 Software, Version 1.1, provided by Roche). The expression data were normalized to the reference glyceraldehyde-3-phosphate dehydrogenase (GADPH). All experiments (sample collection, preparation and storage, primer design, and qPCR normalization) were performed according to the Minimum Information for the Publication of Quantitative Real-Time PCR Experiments (MIQE) guidelines. Three biological repeats were performed for each sample.

### 2.8. Western Blot Analysis

Protein samples were prepared by homogenizing cardiac tissue in radioimmunoprecipitation assay (RIPA) buffer containing proteinase and phosphatase inhibitors (Complete and PhosSTOP; Roche, Basel, Switzerland). Protein extracts from myocardial tissue were separated by 12% sodium dodecyl sulfate-polyacrylamide gel electrophoresis and transferred to 0.22 μm polyvinylidene fluoride membranes (Millipore, Bedford, MA, USA). The membranes were then blocked in Tris-buffered saline with 0.1% Tween 20 containing 5% nonfat dry milk for 2 h at room temperature. Subsequently, the membranes were incubated overnight at 4 °C with primary antibodies, including Nr4a1 recombinant rabbit monoclonal antibody (Cat#MA5-32647, Thermo Fisher Scientific, 1:1000) and HK2 recombinant rabbit monoclonal antibody (Cat#ab209847, Abcam, 1:1000;). The housekeeping protein GAPDH (Cat#KM9002T, Sungene Biotech, 1:4000) was used as reference protein to normalize the target proteins during western blot analysis. After washing, the membranes were incubated with secondary antibodies antibody IgG (Cat# A0208, Beyotime Biotech, 1:1000) at the appropriate dilutions for 1.5 h at room temperature, and detected using the enhanced chemiluminescence substrate kit. The density of each band was quantified using Quantity One. Three biological repeats were performed for each sample.

### 2.9. Statistical Analysis

Values represent the mean ± SD (standard deviation). Statistical significance of differences was determined by Student’s *t*-test or one-way ANOVA with Bonferroni’s post-hoc comparison, where appropriate. Thereby, *p*-values less than 0.05 were considered significant. SPSS Statistics version 26.0 (IBM Corp., Armonk, NY, USA) was used for statistical analysis.

## 3. Results

### 3.1. HFD-Induced Animal Characteristics, Alterations, and cardiac Injury

**Figure 1:**
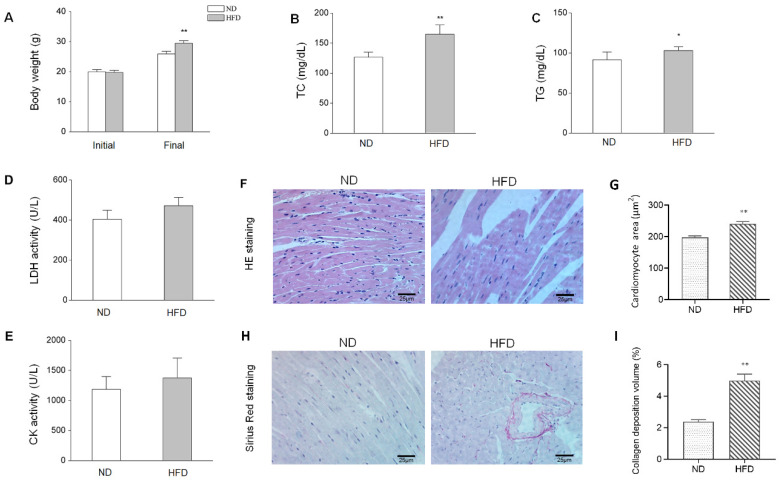
HFD-induced biological characteristics and histopathological alterations. (**A**) Changes in body weight after eight weeks of a normal diet (ND) or high-fat diet (HFD), *n* = 12. (**B**–**C**) Serum levels of TC and TG in mice in the ND and HFD groups. (**D-E**) Alterations of serum LDH and CK activities between groups, *n* = 5. (**F**) H&E staining of myocardium tissues (×400) from ND and HFD groups. (**G**) Quantitative analysis of cardiomyocyte cross-sectional area between groups, *n* = 4. (**H**) Sirius Red staining of the myocardium tissues (original magnification × 400). (**I**) Quantitative analysis of collagen deposition volume between groups, *n* = 4. Data represent means ± SD; * *p* <0.05, ** *p* <0.01, HFD vs. ND group.

After eight weeks of HFD, body weight and other biochemical parameters between different groups were compared to evaluate HFD-induced obesity, which is presented in Figure 1. Mice fed with HFD showed significantly increased body weight, serum TC (*p* < 0.01), and LDH activity (*p* < 0.05), compared with those in the ND group. There also exist raise trend in the content of TG and activity of CK in HFD group compared with ND group, but of not significant (*p* > 0.05). Meanwhile, H&E staining revealed broken fibers, irregular cellular structures and obvious increased cardiomyocyte cross-sectional area (*p* < 0.01) in the left ventricular myocardium of obese mice (Figure 1F–G). HFD also induced collagen deposition volume in the left ventricular myocardium (Figure 1H–I). These results indicate that an obesity mouse model with cardiac injury has been established.

### 3.2. HFD-Induced Alteration of Myocardial Gene Expression

**Figure 2:**
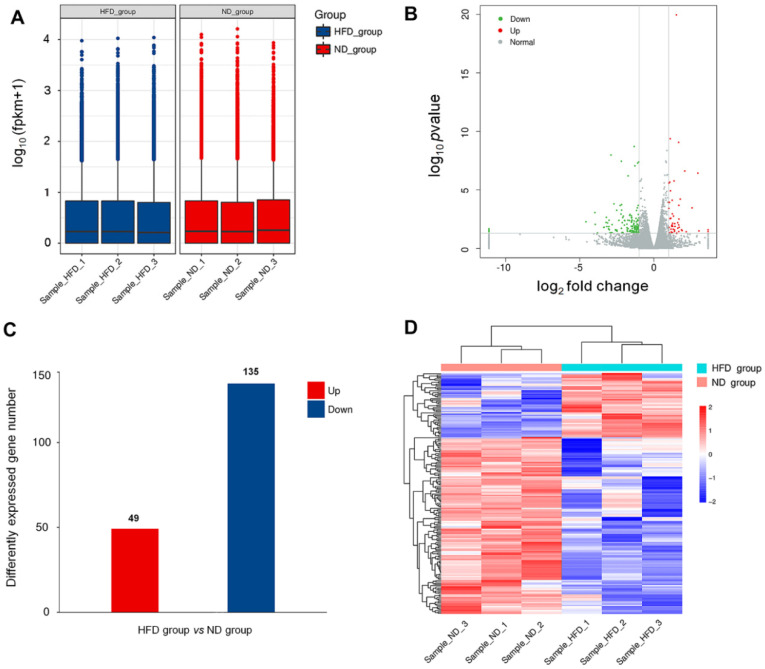
HFD-induced differentially expressed genes (DEGs) in the myocardium. (**A**) FPKM box plot shows the expression levels of transcripts between the ND group and the HFD group. (**B**) Volcano plot showing differential expression with increased DEGs colored in red and decreased DEGs colored in green. (**C**) Altered number of cardiac DEGs induced by HFD. (**D**) Heatmap with hierarchical clustering analyses of DEGs in different samples. The color from blue to red represents the transcript level from low to high.

To investigate the DEGs in obesity-induced cardiac injury, heart tissues were taken from the mice after eight weeks of HFD feeding. Microarray analysis was performed using RNA extracted from respective cardiac samples. The mRNA expression level and abundance were calculated as FPKM values that were applied to develop the box plot (shown in Figure 2A). Transcript analysis of HFD heart tissue revealed a broad spectrum of DEGs. The overall distribution of DEGs is represented in the volcano plot shown in Figure 2B. A corrected *p*-value (*q*-value) < 0.005 and log2 (fold change) >1 were set as the threshold for the DEGs. DEGs analysis showed that a total of 184 genes were differentially expressed (49 increased and 135 decreased) after long-term HFD (Figure 2C). Meanwhile, we performed hierarchical clustering analyses on all transcripts that were differentially expressed, with a heatmap generated to obtain an overview of the differences between the two groups (Figure 2D). As expected, mRNA expression profiles readily separated all samples into two distinct groups based on their feeding status, and samples clustered tightly within each treatment group. In the clustering heatmap, genes with increased expression values are coded from blue to red. Thus, this suggests that the DEGs were altered between HFD and control groups, and limited differences were found within groups.

### 3.3. Functional Annotation of the DEG Profile

**Figure 3:**
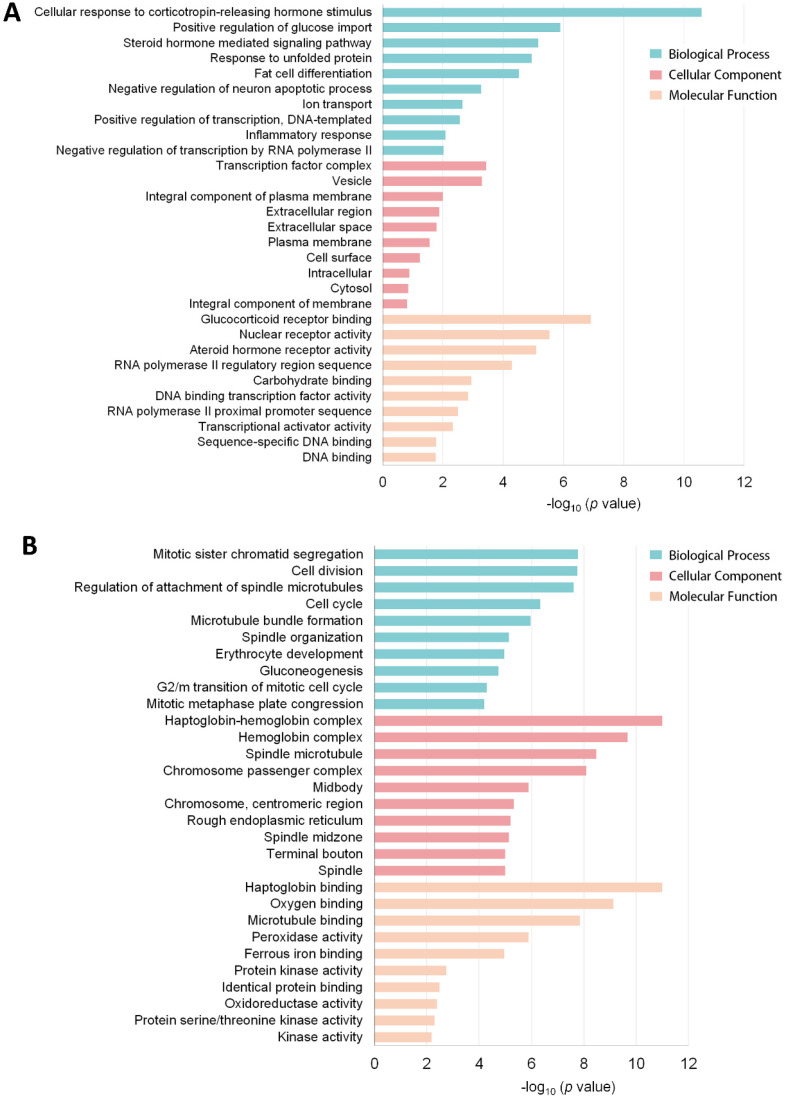
Top 30 GO terms for (**A**) upregulated DEGs and (**B**) downregulated DEGs.

In order to explore the characteristics of DEGs, functional classification of up and downregulated genes was performed using the GO tool based on biological processes (BP), cellular components (CC), and molecular function (MF). GO term analyses of DEGs in heart tissues under obesity conditions clearly reflected the metabolic responses to HFD. As shown in Figure 3A, upregulated DEGs were mainly involved in metabolic homeostasis and hormone-sensing biological processes, including cellular response to corticotropin-releasing hormone stimulus, positive regulation of glucose import, steroid hormone-mediated signaling pathway, response to unfolded protein, and fat cell differentiation. *Nr4a1*, *Nr4a2*, *Nr4a3*, solute carrier family 2 member 3 (*Slc2a3*), and *Irs2* were the critical genes associated with these biological processes. Moreover, the most enriched GO terms for downregulated genes were mitotic sister chromatid segregation, cell division, haptoglobin binding, haptoglobin-hemoglobin complex, chromosome passenger complex, hemoglobin complex, regulation of attachment of spindle microtubules to kinetochores, and oxygen binding (Figure 3B). These GO terms mainly consisted of genes such as *Hbb-bs*, *Hbb-bt*, *Hba-a1*, *Hba-a2*, and *Th*.

### 3.4. KEGG Pathway Enrichment and Network Analysis

**Figure 4:**
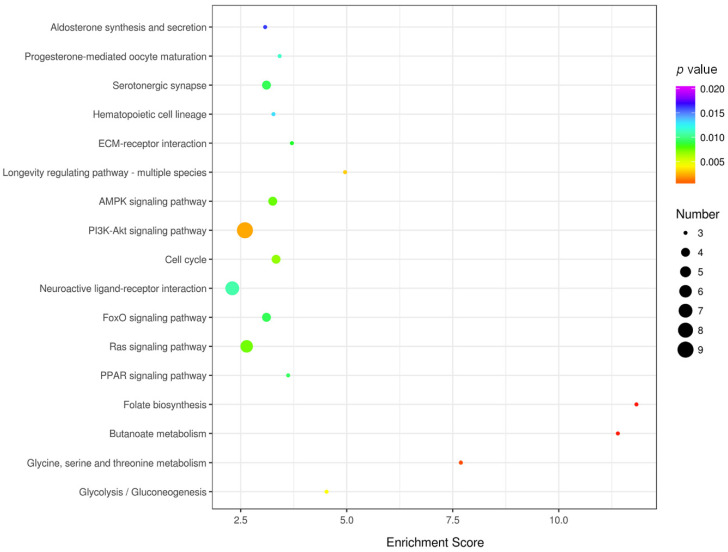
Bubble plot of the significantly enriched KEGG pathways in the HFD group vs. ND group. Colors represent minus logarithms of *p*-values, from purple to red, indicating higher significance. The lengths of the columns represent the numbers of genes enriched in a pathway.

In addition to the GO enrichment analysis, pathway enrichment analysis of DEGs was conducted based on the KEGG pathway database. Pathways with *p*-values < 0.01 were screened out, which indicated a significant contribution to cardiac structural remodeling in obesity. Figure 4 provides an overview of the KEGG pathway enrichment results of DEGs induced by obesity. As observed, folate biosynthesis; butanoate metabolism; glycine, serine, and threonine metabolism; longevity regulating pathway-multiple species; and glycolysis/gluconeogenesis are pathways with relatively high enrichment scores and low *p*-values. Over six DEGs were shown to be involved in the PI3K-Akt signaling pathway, neuroactive ligand-receptor interaction, and Ras signaling pathway. Of all these enriched pathways, downregulated gene sets were mainly involved in glycine, serine, and threonine metabolism; cell cycle; AMPK signaling pathway; PI3K-Akt signaling pathway; and FoxO signaling pathway. The longevity-regulating pathway, MAPK signaling pathway, and PI3K-Akt signaling pathway are the primary enriched pathways of upregulated gene sets.

**Figure 5:**
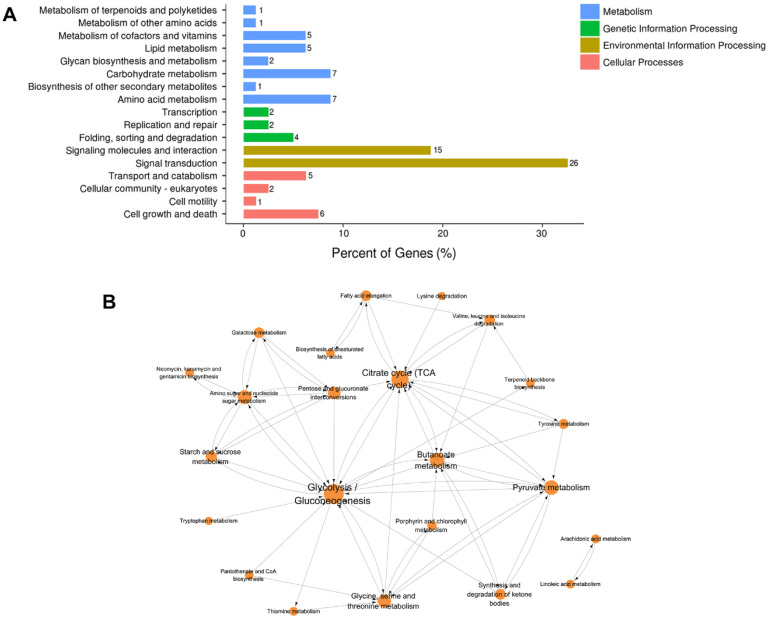
Kyoto Encyclopedia of Genes and Genomes (KEGG) pathway classification and network analysis of enriched genes. (**A**) Enriched KEGG pathway classified into four major levels distinguished by colors. The length of the bar represents the percentage of DEGs involved in secondary KEGG classification. (**B**) The connection of enriched KEGG pathways increased in size, indicating a high degree of certain pathways.

Classification of these enriched pathways was also performed to develop a general and systematic view of the pathways influenced by obesity in the myocardium. This revealed that the classified DEGs were mainly involved in environmental information processing and metabolism (Figure 5A). In environmental information processing, 26 DEGs were related to signal transduction, and 15 DEGs were related to signaling molecules and interaction (detailed information shown in Supplementary Table S2). In addition to the influence on signal pathways, several metabolic pathways were altered by obesity, including carbohydrate metabolism, amino acid metabolism, and lipid metabolism. These results were shown to exacerbate the signal transduction and metabolism dysfunction in the myocardium triggered by long-term HFD. Potential interactions between signal transduction and metabolism may exist, which warrants further investigation.

Moreover, a KEGG pathway network was established to provide an objective perspective of the potential connections between enriched pathways. As shown in Figure 5B, a metabolic pathways network was constructed, which included citrate cycle; glycolysis/gluconeogenesis; pyruvate metabolism; amino sugar and nucleotide sugar metabolism; butanoate metabolism; and glycine, serine, and threonine metabolism. Notably, glucose metabolism-related pathways, presented as the core node, connect these pathways with a high degree, especially glycolysis metabolism, indicating that they may be responsible for HFD-induced myocardium injury. Collectively, KEGG enrichment analysis provides an underlying view of the effects caused by long-term HFD and highlights glucose-related metabolism and signal transduction-related pathways, especially the glycolysis pathway.

### 3.5. Validation of Glucose Metabolism-Related DEGs Using qPCR

**Figure 6:**
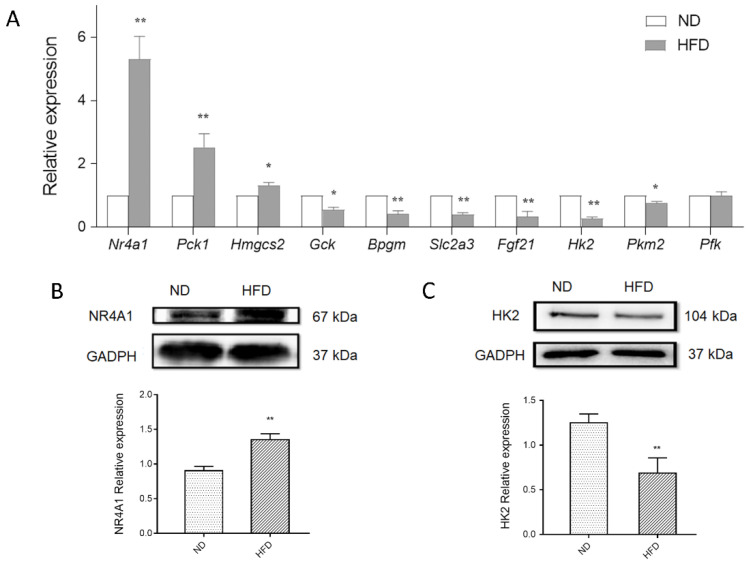
Effect of *Nr4a1* on glucose metabolism homeostasis (**A**) Expression of glucose metabolism related DEGS and key genes in glycolysis metabolism in the myocardium, in response to a normal diet (ND) or high-fat diet (HFD) using qPCR. (**B**,**C**) Protein expression of NR4A1 and HK2 between groups. Data represent means ± SD; *n* = 3, * *p* < 0.05, ** *p* < 0.01, HFD vs. ND group.

Concerning the critical role of glucose metabolism in HFD-induced myocytes, the expression of seven DEGs involved in glycolysis metabolism and glucose signal transduction was determined via qPCR, including fibroblast growth factor 21 (*Fgf21*), soluble carrier family 2 member 3 (*Slc2a3*), *Nr4a1*, phosphoenolpyruvate carboxykinase 1 (*Pck1*), glucokinase (*Gck*), bisphosphoglycerate mutase (*Bpgm*), and 3-Hydroxy-3-Methylglutaryl-CoA Synthase 2 (*Hmgcs2*) (detailed information is shown in Table S3). Consistent with the microarray analysis results, substantially increased levels of *Nr4a1*, *Pck1*, and *Hmgcs2*, and decreased levels of *Gck*, *Bpgm*, *Fgf21*, and *Slc2a3* were observed in the myocardium of HFD mice (Figure 6A). Given the critical role of glycolysis metabolism, the expression of key enzymes in response to HFD stimulation was also detected. Decreased levels of HK2 and PKM2 were observed in the myocardium of HFD mice, especially HK2, the first step-limiting enzyme in glycolysis. In addition, significantly increased NR4A1 (*p* < 0.01) and decreased HK2 (*p* < 0.01) protein levels were observed in myocardium of HFD mice (Figure 6B,C). These results provide evidence of disturbed cardiac glucose homeostasis induced by obesity, ranging from key metabolic genes to signal transduction.

**Figure 7:**
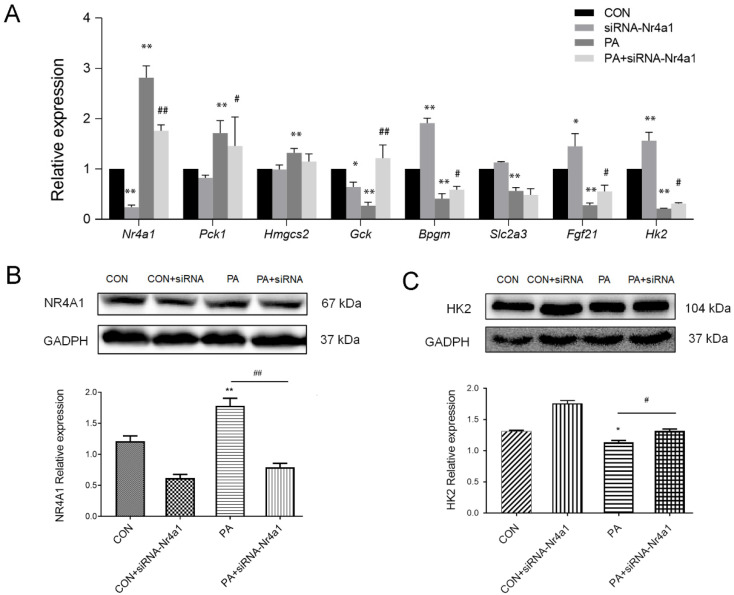
(**A**) PA induction altered expression of DEGs and hexokinase 2 (Hk2) in H9c2 cells, and the effect of knockdown *Nr4a1* by siRNA on the expression of glucose homeostasis-related DEGs under PA treatment conditions. * *p* < 0.05, ** *p* < 0.01, vs. the CON group; ^#^ *p* < 0.05, ^##^ *p* < 0.01, vs. the PA-treated group. (**B**,**C**) *Nr4a1* knockdown significantly decreased the protein level of NR4A1 and increased the protein level of HK2 in H9c2 cells under both regular and PA-treated conditions. *n* = 3, * *p* < 0.05, ** *p* < 0.01, vs. the CON group; ^#^ *p* < 0.05, ^##^ *p* < 0.01, vs. the PA-treated group.

Moreover, in vitro examinations were also carried out to provide further evidence of the molecular alterations in the myocardial cell line caused by obesity. H9c2 cardiac cells were pretreated with PA to induce an insulin-resistant cell model, which is commonly used to mimic in vitro HFD treatment [27,28]. Consequently, similar trends of these DEGs were found in the PA-induced myocardium compared with that in the HFD myocardium (Figure 7A). These results further validate disturbances in glucose metabolism homeostasis in the myocardium triggered by HFD, both in mice and cell lines.

### 3.6. Nr4a1 Regulates the Expression of DEGs in Glucose Homeostasis

Here, we showed that cardiac *Nr4a1* significantly increased in the myocardium of HFD mice and PA-induced H9c2 cells, which may play a role in alterations of hub genes identified in glucose metabolism. We then examined the expression of *Nr4a1* in response to PA-induced impaired glucose homeostasis, which demonstrated that PA caused rapid and robust induction of *Nr4a1* in H9c2 cells (Figure 7A). Knockdown of *Nr4a1* by siRNA-*Nr4a1* under PA treatment conditions, significantly increased glucose metabolism-related DEGs compared to the control, with increased *Fgf21*, *Gck,* and *Bpgm*, and decreased *Pck1* in H9c2 cells. The expression of *Hk2* was also determined under PA or/and siRNA-*Nr4a1* treatment conditions. Consistent with the results in the mouse obesity model, PA treatment significantly decreased *Hk2* in H9c2 cells, which was upregulated by the inhibition of *Nr4a1*. The regulatory effect of Nr4a1 was also observed on the protein expression of HK2 in H9c2 cells (Figure 7B,C). Overall, *Nr4a1* knockdown interrupted PA-induced impaired glucose homeostasis, highlighting the critical role of *Nr4a1* in modulating glucose metabolism homeostasis that is attributed to obesity-induced cardiac injury.

## 4. Discussion

Obesity has reached epidemic proportions worldwide owing to the paradigm shift to a high-calorie diet and a sedentary lifestyle. Moreover, obesity has placed a heavy financial burden on healthcare costs related to numerous pathologies, especially CVD, including coronary artery disease, arrhythmias, heart failure, and sudden cardiac death [29,30]. In the current study, we investigated the global molecular events implicated in the mechanism of cardiac structural remodeling using RNA-Sequencing analysis in an HFD-induced obesity mouse model. The large datasets revealed a global cardiac transcriptome response to HFD challenge; furthermore, valuable genes and pathways implicated in cardiac injuries were identified. In particular, we focused on DEGs involved in glucose metabolism and highlighted the role of NR4A1 in glucose management. These alterations were verified in obesity mouse cardiac tissue and PA-induced H9c2 cardiomyocytes to uncover novel potential targets for the prevention of diet-induced cardiac injuries.

In response to an eight-week HFD, transcriptional profiling data revealed 184 significantly altered genes expressed in cardiac tissues. Functional enrichment analysis revealed that the major biological processes are the cellular response to corticotropin-releasing hormone stimulus, positive regulation of glucose import, and oxygen binding when obesity occurs. These DEGs are mainly implicated in the key components of glucose metabolism, including folate biosynthesis; butanoate metabolism; glycine, serine, and threonine metabolism; and glycolysis/gluconeogenesis metabolism pathways in which were the critical pathways influenced by obesity. Thus, the metabolic dysfunction of myocardium was exacerbated by long-term HFD, especially the dysregulation of glucose metabolism. Obesity causes changes in cardiac glucose metabolism closely connected with the reconstructed energy supply network, which makes ATP production and utilization less efficient. This inefficiency results in functional consequences linked to the increased rate of heart failure in the population [12,31]. During energy metabolism remodeling, increased circulating fatty acids and insulin resistance appeared in response to HFD; accompanied by excess fat storage and inhibition of the metabolic machinery responsible for glucose utilization and oxidation [32]. Consistently, the current cardiac transcriptome analysis revealed dysregulated glucose homeostasis induced by HFD, with *Fgf21*, *Slc2a3*, *Nr4a1*, *Pck1*, *Gck*, *Bpgm*, and *Hmgcs2* serving as the hub genes. These genes are mainly involved in glucose uptake and glycolysis/gluconeogenesis metabolism modulation processes attributed to myocardial glucose homeostasis.

Significant dysregulated glycolysis related genes were observed in obese mice myocardium, especially *Gck*, *Bpgm,* and *Pck1*. *Gck* encodes HK4, which catalyzes the phosphorylation of glucose to glucose 6-phosphate (G6P), thereby serving as the first rate-limiting step in glucose metabolism. Compared to other hexokinases, GCK has a weak affinity for D-glucose and is effective only when glucose is abundant [33]. Generally, GCK is located in the cytoplasm; however, it can shuttle into the nucleus, which may be implicated in gene transcription/new protein synthesis [34]. Bisphosphoglycerate mutase (BPGM) participates in the production of 2,3-bisphosphoglycerate in glycolysis. Furthermore, BPGM regulates hemoglobin oxygen affinity by controlling its allosteric effector, 2,3-bisphosphoglycerate [35]. By managing glycolytic intermediate levels, BPGM thereby modulates downstream processing of glycolysis and serine biosynthetic flux [36]. It has been reported that decreased BPGM was attributed to a failure in the management of glucose homeostasis in acute doxorubicin cardiomyopathy, which may influence the delivery of oxygen to the heart [37]. Phosphoenolpyruvate carboxylase (PCK) participates in the conversion of oxaloacetate to phosphoenolpyruvate in glycogenolysis. With respect to gluconeogenesis, a particular cellular distribution of PCK has been recently described in the liver and kidney of mice and rats and was also observed to be expressed in myocytes [38]. Increased expression of PCK1 in the myocardium implicates the promotion of myocardial glycogenolysis by suppressing glycolysis or glycogen synthesis [39]. Comprehensively, alterations of these hub genes demonstrate the suppression of glycolysis in the myocardium induced by HFD, which is attributed to a disturbance in glucose homeostasis in vivo and in vitro.

Glucose homeostasis is actively orchestrated and carefully choreographed in response to body energy requirements. Maintenance of glucose homeostasis is not limited to the control of metabolic enzymes but is also associated with the regulation of hormones, translational factors, and metabolic intermediates. Glucose transporter-mediated glucose transmembrane uptake serves as the initial step in myocardial glucose metabolism. *Slc2a3*, commonly known as *Glut3*, is expressed in tissues with heightened energy demands and high metabolic rates since it has the highest glucose affinity and greatest transport capacity in the glucose transporter protein family [40]. Hence, it is highly expressed in the brain, cardiomyocytes, and cardiomyoblasts [41]. Decreased expression of *Slc2a3* was observed in the cardiac tissue of HFD mice, indicating dysregulated myocardial glucose transport in response to obesity. The regulator of this process, *Fgf21*, was also identified as a hub gene in obesity-induced cardiac tissue. It has been demonstrated that FGF21, an endocrine hormone, could facilitate glucose uptake by circulating in peripheral tissues [42]. Mice lacking FGF21 are more susceptible to the development of obesity-induced cardiac remodeling [43]. Hepatic-generated FGF21-mediated glucose uptake is dependent on the transcriptional activities of carbohydrate-sensitive transcription factor carbohydrate-responsive element-binding protein (ChREBP) and peroxisome proliferator-activated receptor-α (PPARα), which bind onto the *Fgf21* promoter to induce its expression [44]. The activation of ChREBP was in response to the content of the glycolysis intermediate G6P in the liver; hence, forming a feedback regulation between *Fgf21* and glucose metabolism via glycolysis [45]. Here, we observed significant alterations in *Gck* and *Fgf21* expression, indicating a severely disturbed glycolysis metabolism in the myocardium, which was stimulated by obesity.

Evidence in the current study identified *Nr4a1* as the critical regulator of cardiac glucose homeostasis in response to nutrient stimulation. Here, we showed that the expression of cardiac *Nr4a1* significantly increased in the myocardium of HFD mice and PA-treated H9c2 cells, which may result in alterations of DEGs in glucose metabolism. However, inhibition of *Nr4a1* could enhance glycolysis by enhancing the expression of the key step genes, such as *Gck*, *Bpgm*, *Pck1*, *Hk2*, and *Pkm2*, as well as modulating *Fhgf21* mRNA. Consistent with our results, *Nr4a1* promotes gluconeogenesis and suppresses glycolysis by attenuating sumoylation-regulated *Pck* stability in an acetylation-dependent manner in human hepatoma cell lines [46]. As a transcription factor, *Nr4a1* can bind to the promoter region of the Nur77-binding response element of glycogenolysis and glucose uptake-related genes in muscle, thereby regulating glucose metabolism [47]. Recently, considerable attention has been paid to its function in the cardiovascular system, but limited mechanisms have been obtained. Cardiac overexpression of *Nr4a1* promotes a disturbance in cardiomyocyte calcium homeostasis and adverse cardiac remodeling, depending on the glucose regulation mechanism [22,48]. *Nr4a1* could negatively regulate cardiac contractility via activation of the hexosamine biosynthetic pathway with downstream effects on protein O-linked glycosylation in the myocardium. As we described, knockdown of *Nr4a1* in H9c2 cells resulted in upregulated levels of HK2, suggesting that *Nr4a1* may function upstream of HK2, or have an effect on the transcription and translation process of HK2; therefore, handling glycolysis and glucose metabolism homeostasis. These data indicate that *Nr4a1* is implicated in cardiac glucose homeostasis, closely linked to the regulation of the hub glucose metabolic genes, serving as a promising target in obesity-induced cardiac impairment. However, specific molecular mechanisms still need further investigation.

## 5. Conclusions

In the current study, a high-throughput sequencing approach was employed to illustrate the critical cardiac alterations of molecular events in HFD-induced cardiac injuries. Based on bioinformatic analysis, it was observed that the 184 DEGs were mainly implicated in hormone sensing and metabolic homeostasis biological processes, particularly enriched in signal transduction and glucose metabolism pathways. The expression of DEGs involved in glucose metabolism pathways was validated both in the myocardium of obese mice and PA-treated H9c2 cardiomyocytes by qPCR, with consistent obtained change trends. *Nr4a1* was addressed owing to its significantly increased myocardium expression in response to HFD, and its potential role as a glucose metabolism homeostasis regulator. Additionally, in vitro blockage of *Nr4a1* could partially promote glycolysis metabolism by activating the rate-limiting enzyme HK2 in PA-induced dysregulated glucose homeostasis. Our transcriptome analysis provides a comprehensive understanding of the molecular genetic framework for how HFD influences the cardiac transcript profile during the progression of obesity. Furthermore, we propose that *Nr4a1* may be a promising therapeutic target for preventing obesity-induced CVD. Further unbiased research is needed on the molecular mechanisms by which *Nr4a1* handles myocardial glucose metabolism homeostasis and subsequently controls cardiac functions in obesity-related cardiovascular pathophysiology.

## Supplementary Materials

The following are available online at https://www.mdpi.com/2073-4425/11/7/720/s1, Table S1: Information of Primers applied in qRT-PCR, Table S2: Classification of the enriched KEGG pathways, Table S3: Information of Glucose Metabolism Related DGEs.
